# Chemical Modification of Bacterial Cellulose for the Development of an Antibacterial Wound Dressing

**DOI:** 10.3389/fbioe.2020.557885

**Published:** 2020-09-24

**Authors:** Isabel Orlando, Pooja Basnett, Rinat Nigmatullin, Wenxin Wang, Jonathan C. Knowles, Ipsita Roy

**Affiliations:** ^1^School of Biosciences, College of Liberal Arts and Sciences, University of Westminster, London, United Kingdom; ^2^School of Medicine and Medical Sciences, Charles Institute of Dermatology, University College Dublin, Dublin, Ireland; ^3^Université Clermont Auvergne, Centre Nationale de la Recherche Scientifique (CNRS), SIGMA Clermont, Institut de Chimie de Clermont-Ferrand (ICCF), Clermont–Ferrand, France; ^4^Advanced Composites Collaboration for Science and Innovation, University of Bristol, Bristol, United Kingdom; ^5^Division of Biomaterials and Tissue Engineering, University College London (UCL) Eastman Dental Institute, London, United Kingdom; ^6^Department of Nanobiomedical Science and BK21 Plus NBM, Global Research Center for Regenerative Medicine, Dankook University, Cheonan, South Korea; ^7^The Discoveries Centre for Regenerative and Precision Medicine, University College London, London, United Kingdom; ^8^Department of Materials Science and Engineering, Faculty of Engineering, University of Sheffield, Sheffield, United Kingdom

**Keywords:** bacterial cellulose, antibacterial, wound dressing, green chemistry, biodegradable polymers

## Abstract

Bacterial cellulose is a bacterially derived polymer with great potential for application in wound healing due to its innate properties such as high biocompatibility and biodegradability. In addition to this, it is naturally biosynthesized by bacteria as a hydrogel, which makes it an optimal substrate for the treatment of dry wounds, where additional moisture is required to facilitate the healing process. However, this polymer lacks antibacterial properties. As bacterial infections are becoming increasingly common and difficult to treat due to antimicrobial resistance, it is of crucial importance to develop strategies for the modification of cellulose to ensure protection against bacterial contamination. In this study, a green-chemistry approach was proposed for the functionalization of cellulose to introduce antibacterial functional groups. Two different active agents, namely glycidyl trimethylammonium chloride and glycidyl hexadecyl ether, were used for the covalent derivatization of the hydroxyl groups of glucose through a heterogeneous reaction in basic aqueous conditions. The modified material was chemically and mechanically characterized by solid-state techniques and rheological measurements. A biological assessment was then carried out both using bacterial cells and human keratinocytes. It was observed that the functionalization performed induced a reduction of approximately half of the bacterial population within 24 h of direct contact with *Staphylococcus aureus* subsp. aureus Rosenbach 6538P^TM^ and *Escherichia coli* (Migula) Castellani and Chalmers ATCC^®^ 8739^TM^ (respectively, a reduction of 53% and 43% in the cell number was registered for the two strains). In parallel, cytotoxicity studies performed on keratinocytes (HaCaT cell line) showed cell viability in the range of 90 to 100% for up to 6 days of direct contact with both unmodified and modified samples. The morphology of the cells was also visually evaluated, and no significant difference was noted as compared to the control. Finally, the *in vitro* scratch assay evidenced good wound closure rates in the presence of the samples, with complete coverage of the scratched area after 5 days for both the modified cellulose and the positive control (i.e., keratinocytes growth medium). Overall, the modified hydrogel showed promising features, confirming its potential as an alternative substrate to develop a sustainable, antibacterial and biocompatible wound dressing.

## Introduction

Over the last decades, the exceptional intensification of antimicrobial resistance (AMR) has led the scientific community to allocate time and money to researching alternative strategies to fight the spreading of infections. Resistance mechanisms started to arise soon after antibiotic discovery, but only with their worldwide distribution and availability at low prices they have come to pose a real threat to human health (Walsh, 2000; Tenover, 2006). It is in fact generally acknowledged that the overuse of antibiotics, both in developed and developing countries (where they can be purchased over the counter), has triggered bacteria to develop resistance, not only because of the over-exposure but also as a result of their consumption in wrong dosages (Reardon, 2014). In addition to this, the void in the discovery of new classes of antibiotics for the last 30 years has contributed to an increase in the mortality rate (The PEW charitable trust, 2016). It has been estimated that 700,000 people die every year as a result of infections and malaria, and the number is likely to increase over the next years (O’Neill, 2018). One major medical area affected by AMR is wound healing. Wounds can in fact provide an access for bacteria to the inner tissues and organs, thus posing a high risk of bloodstream infections that can lead to morbidity. An example of wound-related infections is represented by surgical site infections (SSIs), i.e., infections contracted within the first 30 days of surgery. SSIs account for about 20% of all the healthcare associated infections and increase the risk of death in post-operative patients by 2–11 times. SSIs also prolong the hospitalization time, resulting in higher costs for health-care institutions (Anderson et al., 2014).

In this context, the need for alternative materials that can effectively inhibit bacterial growth without triggering resistance has become urgent. One of the most important classes of biomedical materials is represented by polymers. In particular, the use of natural polymers has recently increased thanks to their specific features. These polymers are in fact characterized by a high degree of biocompatibility, mostly due to their chemical structures that resemble that of the extracellular matrix components. Furthermore, such structures are often difficult if not impossible to replicate through synthetic pathways, making these polymers a precious resource for various biomedical applications (Aravamudhan et al., 2014; Olatunji, 2016). Bacterial cellulose (BC) is one of the most studied natural polymers thanks to its unusual characteristics. This polymer is naturally produced by fermentation of various bacteria, although there are examples in the literature of self-assembly of BC through cell-free methodologies based on the use of *in vitro* enzymatic systems (Ullah et al., 2015; Kim et al., 2019). Among the different BC-producing strains, *Gluconacetobacter xylinus* (formerly *Acetobacter xylinum*) is the most commonly used due to its high polymer production yield and the wide range of carbon and nitrogen sources that it can utilize (Chawla et al., 2009). The average degree of polymerization for this strain has been found to be in the range between 2190 and 3470 with peaks of 6000 after 5 days, although it has been reported that the molecular weight shows a linear increase with the generation change of bacteria (Legge, 1990; Okajima et al., 1991; Iguchi et al., 2000). The biosynthesis of BC involves the incorporation of huge quantities of water in the macromolecular structure, resulting in the formation of a natural hydrogel with over 90% of its total mass consisting of water. This is a crucial parameter for wound healing, as dry wounds require additional moisture to ensure the regeneration of new tissues and to avoid necrosis. These wounds are in fact characterized by the presence of devitalized necrotic tissue, which gives them a black color. Such a feature is a consequence of dehydration and cell death, and it has been observed that the removal of necrotic tissue (for instance through excision) is necessary to promote wound healing (Abdelrahman and Newton, 2011; Kavitha, 2014). However, when surgery is not possible due to the patient’s conditions or if the amount of necrotic tissue is too low, alternative solutions can be adopted such as the use of hydrogels as wound dressings, which can facilitate the autolytic debridement of necrotic tissue (Abdelrahman and Newton, 2011; Dhivya et al., 2015). BC is also characterized by higher purity as compared to plant-derived cellulose, for which harsh chemical treatments are necessary to remove other wood-pulp components such as hemicellulose and lignin (Helenius et al., 2006). Additionally, its nanofibrillar structure gives this biopolymer high porosity and surface area, resulting in improved water-holding capacity as compared to plant cellulose. BC is also characterized by higher crystallinity and higher mechanical properties than the plant-derived one, such as better tensile strength and Young’s modulus (Picheth et al., 2017; Ul-Islam et al., 2019a). For these reasons, BC is particularly suitable for applications in the biomedical field and, more specifically, for skin tissue engineering and wound healing. However, due to its lack of antibacterial activity, chemical or physical modifications are needed to ensure protection against bacterial contamination.

In the field of wound healing, several approaches have been investigated, often involving the absorption of external agents that are released from the polymer matrix over time. A very common example of active compounds is represented by metal ions. In this context, silver is probably the most common metal, and it has been incorporated into BC in different forms such as salts (Chen et al., 2019), bound to zeolites (Gupta et al., 2016) or montmorillonite (Horue et al., 2020), silver-based fluorescent complex (namely, [Ag(ImD)_2_]ClO_4_) (DeBoer et al., 2015) and silver sulfadiazine (Faisul Aris et al., 2019). Other metals have also been explored for this purpose, in particular, the introduction of zinc and zinc oxide has been investigated (Janpetch et al., 2016; Shahmohammadi Jebel and Almasi, 2016; Khalid et al., 2017; Wahid et al., 2019a; Dharmalingam and Anandalakshmi, 2020; Dincă et al., 2020) as well as titanium oxide (Khan et al., 2015; Ullah et al., 2016). In addition to this, organic compounds have been used, including natural extracts (Siddhan et al., 2016) and antibiotics (Shao et al., 2016). Although this methodology often allows to develop efficient biomaterials through easy processes, there are a few drawbacks related to the use of leachable agents, such as the maximum amount that can be loaded into the matrix, the stability of low molecular weight compounds, which are often characterized by higher reactivity and volatility (for instance, in the case of antibacterial natural compounds) and the short-term activity, which is dependent on the release profile of such agents (Giano et al., 2014; Deka et al., 2015). Another strategy for the development of BC-based antibacterial materials involves the combination of BC with antibacterial polymers such as chitosan (Lin et al., 2013; Kingkaew et al., 2014; Wahid et al., 2019b; Yin et al., 2020) and ε-polylysine (Gao et al., 2014). In this case, however, it is fundamental to ensure good compatibility between the two components to avoid phase separation. To overcome these issues, a chemical modification can be performed to impart long-term and structural-related activity to the system.

In this study, BC was chemically functionalized following a green-chemistry approach to introduce antibacterial functionalities. In particular, its surface hydroxyl groups were derivatized under heterogeneous conditions in water. For this purpose, two epoxides containing, respectively, a quaternary ammonium group and an alkyl chain were subjected to base-catalyzed ring-opening reaction. Quaternary ammonium groups are widely used substrates for the development of biocides utilized in various fields, including water disinfection (Abid et al., 2010; Farah et al., 2015), textiles (Nayak and Padhye, 2015) and biomedical applications such as dental (Antonucci et al., 2012; Cheng et al., 2012), orthopedic (Tan et al., 2013), and wound healing (Fan et al., 2015; Liu et al., 2018). Their structure presents a cationic nitrogen that allows the adsorption of the compound onto the negatively charged portions of the bacterial membrane. The mechanism of action of quaternary ammonium groups is believed to involve membrane damage, with consequent alteration of the bacterial equilibrium and leakage of intracellular constituents. After a first attraction, the hydrophobic tails of the compound are in fact able to penetrate through the lipid regions of the membrane, causing its disruption and, ultimately, cell death (Ioannou et al., 2007). Along with cationic ammonium groups, alkyl chains (C_14_) were attached to the polymer backbone. Similarly to the mechanism described for quaternary ammonium groups, these structures can also interact hydrophobically with the bacterial cell wall and induce cell death (Denis et al., 2010; Wiener and Horanyi, 2011; Zheng et al., 2016). A fundamental parameter to be considered, however, is the length of the chain. It has been found that the optimum can vary for Gram-positive and Gram-negative strains, although generally higher activity is achieved for lengths higher than C_12_, whereas no antibacterial effect has been observed below C_4_, probably due to the inability of crossing the hydrophobic region of the membrane (Devínsky et al., 1990; Zhao and Sun, 2008). After the reaction, the material obtained was chemically and mechanically characterized, and its features were compared to unmodified BC. A biological assessment was then conducted toward both bacterial and human cells to determine the antibacterial activity and the cytotoxicity of the hydrogels as well as their suitability as wound healing platforms.

## Materials and Methods

### Materials

Dulbecco’s modified Eagle medium (DMEM), fetal bovine serum (FBS), sodium pyruvate, penicillin/streptomycin, 0.25% trypsin/ethylenediaminetetraacetic acid (EDTA), trypan blue, Alamar Blue, rhodamine phalloidin and 4’,6-diamino-2-phenylindole, dilactate (DAPI) were purchased from Thermo Fischer Scientific Inc. Sodium hydroxide was purchased from VWR International. All the other chemicals and reagents used in this work were purchased from Sigma-Aldrich (now Merck kGaA). Gram-negative bacterium *Gluconacetobacter xylinus* JCM10150 used to produce bacterial cellulose was obtained from the culture collection of the University of Westminster, London, United Kingdom. The antibacterial assays described were performed using Gram-positive *Staphylococcus aureus* subsp. aureus Rosenbach 6538P^TM^ and Gram-negative *Escherichia coli* (Migula) Castellani and Chalmers ATCC^®^ 8739^TM^, both purchased from ATCC^®^. The cytotoxicity studies were carried out using *in vitro* spontaneously transformed keratinocytes (HaCaT) from histologically normal skin (catalog number: T0020001), bought from AddexBio.

### Bacterial Cellulose Production and Purification

Bacterial cellulose was produced by *Gluconacetobacter xylinus* using glucose as the main carbon source. In particular, a modified Hestrin and Schramm medium was used as the production medium, which contained glucose (50 g/L), yeast extract (5 g/L), and CaCO_3_ (12 g/L). The final pH was adjusted to 5.5 using 1 M HCl and 1 M NaOH. The medium constituents were sterilized in an autoclave at different temperatures depending on their nature to avoid thermal degradation of the sugar. Pre-inoculation was then carried out using frozen bacterial glycerol stocks and 10 mL of production medium into 20 mL glass vials. The vials were kept slightly open to ensure sufficient air flow, although filters or cotton caps might also be used to minimize the risk of cross-contamination. After formation of the pellicle, 3 mL of inoculum were transferred into 500 mL Erlenmeyer flasks containing 300 mL of sterile production medium. Static fermentation was performed at 30°C for 5–7 days or until pellicles with a thickness of 1–1.5 cm were obtained. The membranes were then harvested and washed several times using distilled water. In order to remove residual biomass, cellulose pellicles were treated with 1 M NaOH for 2 h at 80°C under continuous agitation and washed with distilled water until neutral pH was achieved. The process was repeated twice. Prior to their use, the pellicles were sterilized in the autoclave at 110°C for 10 min.

### Evaluation of Water Content

The water content of the cellulose pellicles was determined through evaluation of their weight before and after a drying period of 24 h under a fume-hood. 9 samples from three different pellicles were used. The percent of water was calculated using the following formula:

$$\mathrm{Water}\mathrm{content}\%=\frac{A-B}{A}\times 100$$

*A* = Weight of the wet sample.

*B* = Weight of the dry sample.

### Chemical Functionalization

The surface of bacterial cellulose membranes was functionalized using two different epoxides containing quaternary ammonium groups and hydrophobic tails. The reaction was carried out in aqueous system. First, 5% w/w NaOH with respect to cellulose (dry) was used for the deprotonation of the hydroxyl groups of glucose. After 15 min of basic pre-treatment of the samples at room temperature, glycidyl trimethylammonium chloride (GTMAC) and glycidyl hexadecyl ether (GHDE) were added in excess to the stoichiometric ratio. The stoichiometric ratio was calculated considering that each monomeric unit of cellulose (i.e., glucose) presents three hydroxyl groups. The reaction was performed at 65°C in a water bath for 4 h under continuous stirred conditions. The samples were then washed with distilled water for several hours until neutral pH was reached.

### Morphological Analysis

The morphology of the pellicles was studied through Scanning Electron Microscopy on dry samples after evaporation for 24–48 h (depending on the thickness) under a fume-hood. Non-modified and modified cellulose structures were observed using a JOEL 5610LV scanning electron microscope. The samples were placed on the 8 mm diameter aluminum stubs and gold-coated for 2 min using the gold sputtering device (EMITECH-K550). Operating pressure of 7 × 10^–2^ bar and deposition current of 20 mA (for 2 min) were used. The analysis was carried out at the Eastman Dental Institute, Department of Biomaterials and Tissue Engineering, University College London, United Kingdom.

### Chemical Characterization

The chemical structure of the pellicles before and after modification was also investigated. Prior to each analysis, the samples were dried for 24–48 h under a fume-hood. The same equipment described in section “Morphological Analysis” was used to carry out an elemental analysis through Energy-dispersive X-ray spectroscopy (EDX). Chemical characterization of the polymer was also performed by X-ray photoelectron spectroscopy (XPS) using a Thermo Escalab 220iXL system. The analysis was conducted with an Al K_*α*_ mono-chromated X-ray source and data were elaborated using CASAXPS (Casa Software Ltd, Teignmouth, United Kingdom). For all the samples, both survey and high-resolution spectra were recorded. The measurement was carried out at the School of Chemistry, Cardiff University, United Kingdom.

### Rheological Studies

The viscoelastic behavior of the cellulose membranes was determined through rheological measurements using a Discovery HR-2 Rheometer (TA Instruments). The test was performed on 1 cm^2^ samples of non-modified cellulose and cellulose after modification. A parallel plate geometry with a diameter of 8 mm was used and the gap was set at 3.85 mm for all the measurements. First, the linear viscoelastic region (LVR) was determined through oscillation sweep test by applying increasing amplitude stress (0.01–100 Pa) and constant frequency (1.0 Hz). All the other assays were conducted using a stress within the LVR. Frequency sweep analysis was carried out to measure the variations of storage modulus and loss modulus for each cellulose type. Stress of 6 Pa, temperature of 32°C and increasing frequency from 0.1 to 50 Hz were applied. The influence of temperature on the storage modulus and loss modulus was also evaluated through temperature ramp assay in the range between 10 and 60°C with an increasing rate of 5°C/min. The test was performed using a stress of 6 Pa and a frequency of 1 Hz.

### Stability of Bacterial Cellulose

The stability of the cellulose pellicles in aqueous media was studied for 7 days. Three samples of 1 cm^2^ were used for both unmodified and modified cellulose in order to investigate the effect of the reaction performed. The weight variation was registered both in phosphate buffer solution (PBS) and keratinocytes growth medium through evaluation of the initial hydrated weight and the hydrated weight of each sample after 1, 3, 5, and 7 days. The specimens were placed in 24-well plates with 2 mL of medium in each well and incubated in static conditions at 32°C. The percent weight variation (ΔW) for each time point was calculated using the formula below:

$$\text{Δ}W\%=\frac{W_{x}}{W_{0}}\times 100$$

W_x_ = Weight of the sample at time x.

W_0_ = Weight of the sample at time 0.

### Antibacterial Activity Evaluation

#### Bacterial Culture Methods

The antibacterial tests were carried out using two strains, i.e., *Staphylococcus aureus* subsp. aureus Rosenbach 6538^TM^ and *Escherichia coli* (Migula) Castellani and Chalmers ATCC^®^ 8739^TM^ (from here onwards referred to as, respectively, *S. aureus* and *E. coli*). Selective mannitol agar was used as the solid culture medium for *S. aureus*, while MacConkey agar was used for *E. coli*. The plates were prepared by streaking a loopful of inoculum from the glycerol stocks and kept in the incubator at 37°C under static conditions overnight. The culture was prepared through inoculation of 150 mL of nutrient broth No. 2 in a 500 mL Erlenmeyer flask using a single colony. The inoculum was incubated at 37°C in shaking conditions at 120 rpm until log phase (approximately 16 h). The optical density (OD) was then measured at 600 nm and the culture was diluted to 0.5 MacFarland concentration (i.e., 1.5 × 10^8^ CFU/mL), which corresponds to OD_600_ = 0.132. The assay was performed through dilution of such inoculum using nutrient broth diluted 1:500 until the appropriate cell concentration was reached.

#### Direct Contact Test (DCT)

The antibacterial properties of modified cellulose were studied by adapting an ISO 22196 procedure for the quantitative evaluation of the activity upon direct contact. As indicated in the protocol, untreated cellulose was used as the control. Three 2.5 cm^2^ samples of functionalized cellulose as well as six 2.5 cm^2^ samples of non-functionalized cellulose were sterilized in an autoclave and placed onto agar plates. 100 μL of *S. aureus* or *E. coli* inoculum at cell concentration of 1.2 × 10^6^ CFU/mL were pipetted directly on the surface of each sample. Three of the unmodified pellicles as well as three of the modified ones were incubated under static conditions at 37°C for 24 h, while the remaining three samples of unmodified cellulose were washed to quantify the number of bacterial cells recovered at time 0. For the recovery of bacteria, the samples were transferred to a sterile glass vial containing a known amount of phosphate buffer saline (PBS) solution and the bacteria were detached through vortexing of the mixture for 3 min. Several 10-fold dilutions were prepared using PBS. 10 μL of each dilution were then plated on nutrient agar plates through drop plate technique. When the drops were completely dry, the plates were placed upside down in the incubator at 37°C under static conditions for 20 h. The same method was used for the recovery of bacteria at 0 and 24 h. The number of viable cells was evaluated by colony counting. The antibacterial activity was then calculated using the following formula:

$$R\%={\frac{\left(a\right)\left[unmodified\right]-\left(a\right)\left[modified\right]}{\left(a\right)\left[unmodified\right]}}^{*}100$$

where *a* is the average of the viable bacterial cells recovered after 24 h expressed as CFU/(mL^∗^cm^2^).

### Biocompatibility Assessment

#### Cell Culture Methods

Keratinocyte (HaCaT cell line) cells were cultured in T75 flasks using Dulbecco’s modified Eagle medium (DMEM) supplemented with 10% v/v fetal bovine serum (FBS), 1% v/v sodium pyruvate and 1% v/v penicillin/streptomycin and incubated at 37°C in 5% CO_2_ atmosphere. Upon reaching a confluency of 80–90%, cells were sub-cultured with a 1:5 ratio through incubation for 3 min with 3 mL of 0.25% trypsin/ethylenediaminetetraacetic acid (EDTA). The reagent was then inactivated by addition of 12 mL of serum-supplemented medium. The cells were centrifuged at 120 x g for 5 min, and the pellet obtained was resuspended using a known volume of medium. The count of the cells was carried out using a Neubauer counting chamber by trypan blue exclusion method.

#### Indirect Cytotoxicity Assay

For the evaluation of the indirect cytotoxicity, 1 cm^2^ samples of non-functionalized and functionalized cellulose in triplicate were sterilized in autoclave and incubated in a 24-well plate with 1 mL of growth medium for 24 h at 37°C in 5% CO_2_ atmosphere. On the same day, 5 × 10^4^ cells per well were seeded in a 24-well plate and incubated with 1 mL of growth medium in the same conditions. After 24 h, the medium was removed and replaced with conditioned medium. Non-conditioned medium was used as the positive control, whereas pure dimethyl sulfoxide (DMSO) was used as the negative control. The cells were incubated with the eluates for 24 h at 37°C in 5% CO_2_ atmosphere. On the following day, the medium was removed and 1 mL of fresh medium containing 10% v/v of Alamar Blue was added to each well. The plate was then incubated in the same conditions for 4 h. After this time, 200 μL of the solution from each well were transferred into a 96-well plate. The absorbance was measured at 570 nm and normalized to the absorbance at 600 nm using a FluoStar Optima plate reader (SMG Labtech). The cell viability was then calculated through comparison of each value to the positive control using the formula below:

$$\mathrm{Cell}\mathrm{viability}=\frac{Ax}{Ap}\times 100$$

A_x_ = absorbance of the solution – absorbance of TCP/10% Alamar Blue.

A_p_ = absorbance of the positive control – absorbance of TCP/10% Alamar Blue.

#### Direct Cytotoxicity Assay

The cell viability after direct contact with functionalized cellulose was evaluated following the ISO 10993-5 procedure for the biological characterization of medical devices. 1 cm^2^ samples of non-functionalized and functionalized cellulose in triplicate were sterilized in an autoclave and pre-conditioned with 1 mL of growth medium for 24 h at 37°C in 5% CO_2_ atmosphere. On the same day, 5 × 10^4^ cells per well were seeded in a 24-well plate and incubated with 1 mL of growth medium under the same conditions. After 24 h, the medium was removed from the cells and the samples were placed directly onto the cell layer. 1 mL of fresh medium was added into each well. Growth medium was used as the positive control and pure DMSO was used as the negative control. The cells were incubated with the samples at 37°C in a 5% CO_2_ atmosphere. Cell viability was determined after 1, 3, and 6 days through Alamar Blue assay following the procedure described in section “Indirect Cytotoxicity Assay.” After 6 days of contact with the samples, the cells were stained and their morphology was visually evaluated. Two stains were used, i.e., rhodamine phalloidin and 4’,6-diamino-2-phenylindole, dilactate (DAPI). First, the cells were fixed with a 4% formaldehyde-based fixation buffer and incubated for 30 min at room temperature. After this, the excess buffer was removed by washing with PBS solution. 0.1% Triton X-100 in PBS was then added to increase the permeability, incubated for 3–5 min and washed with PBS three times. Finally, the staining solution containing 8 μL/mL rhodamine phalloidin and 1 μL/mL DAPI was added to the cells. The images were acquired with a confocal microscope, Leica TCS SP2.

#### *In vitro* Scratch Assay

The migratory and proliferative ability of keratinocytes in the presence of the cellulose samples were evaluated through *in vitro* scratch assay. 1 cm^2^ samples in triplicate of non-functionalized and functionalized cellulose were sterilized in an autoclave and pre-conditioned with 1 mL of growth medium for 24 h at 37°C in 5% CO_2_ atmosphere. On the same day, 1 × 10^5^ cells per well were seeded in a 24-well plate and incubated with 1 mL of growth medium in the same conditions. After 24 h, the medium was removed from the cells and a scratch was made in the cell monolayer using the tip of a P200 pipette. The cells were then washed with 1 mL of PBS solution to remove the cell debris and a reference mark was made next to the scratch with an ultrafine tip marker. The cellulose samples were then placed directly onto the cell layer together with 1 mL of fresh medium. Growth medium was used as the positive control, whereas pure DMSO was used as the negative control. At time 0, the width of the scratch was set as 100% and the scratch coverage by cells at 0%. The progressive coverage of the scratched area by cells was observed using an Olympus CKX41 microscope, bright field, magnification 40× and 100×. The images were acquired with a QIClick^TM^ CCD Camera from QImaging. The pictures were adjusted using GIMP, an open source software: the contrast was minimized and the brightness was increased in order to have a clear distinction between the cells (white pixels) and the scratch (black pixels). The analysis of the image was carried out with ImageJ (National Institutes of Health, United States). The percentage of coverage of the scratch was considered as the ratio between white pixels and black pixels in that area and was monitored over time through quantification of the black pixels in the image.

## Results

### Bacterial Cellulose Production

**FIGURE 1:**
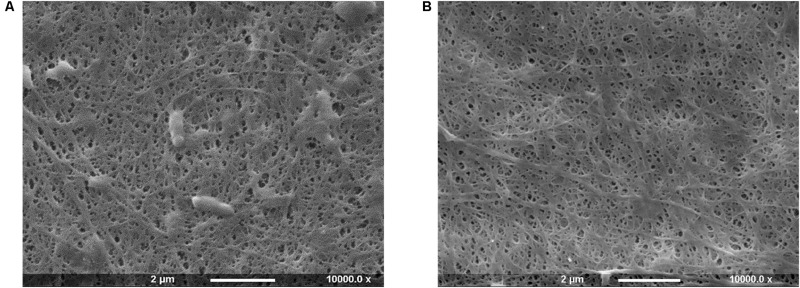
SEM images of bacterial cellulose pellicles before **(A)** and after **(B)** purification treatment.

Bacterial cellulose is secreted extracellularly by bacteria in the form of glucan chains that self-assemble into microfibrils immediately outside the cell membrane (Chawla et al., 2009; McCarthy et al., 2019). As a result, *Gluconacetobacter xylinus* cells remain trapped within the highly porous structure in the interstices between the fibrils. In light of this, a high-temperature basic treatment was carried out to ensure the complete removal of the biomass (Figure 1).

The SEM analysis of the pellicles before purification showed the presence of cells in the network, whereas after the treatment bacteria appeared to be successfully removed without significant degradation or alteration of the arrangement of the chains and the supramolecular structure of cellulose.

The water content of the pellicles produced was also evaluated. The difference in the weight of cellulose samples was evaluated before and after drying under a hood for 24 h. As expected, the pellicles presented an average 97% w/w of water, confirming the hydrogel nature of the biomaterial. The moisture incorporated during the synthesis was in fact retained and stabilized by the highly porous network, which turns BC into an optimal substrate for applications on dry wounds. Moreover, the drying time was found to be much longer when exposed to air, with a total of 4–5 days required for dehydration, which is beyond the range of the average dressing change period for hydrogels, i.e., 1–3 days (Purser, 2010; Sood et al., 2014).

### Chemical Functionalization

**FIGURE 2:**
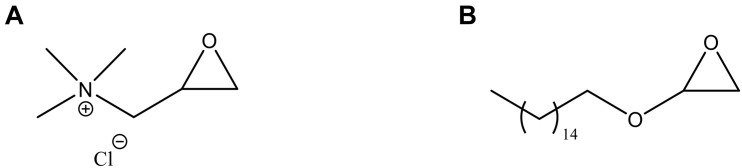
Chemical structure of **(A)** GTMAC and **(B)** GHDE.

As cellulose lacks antibacterial features, the pellicles produced were subjected to chemical treatment in order to incorporate active functional groups through covalent attachment to the polymer backbone. More specifically, two different epoxides were used for this purpose, namely glycidyl trimethylammonium chloride (GTMAC) and glycidyl hexadecyl ether (GHDE) (Figure 2).

**FIGURE 3:**
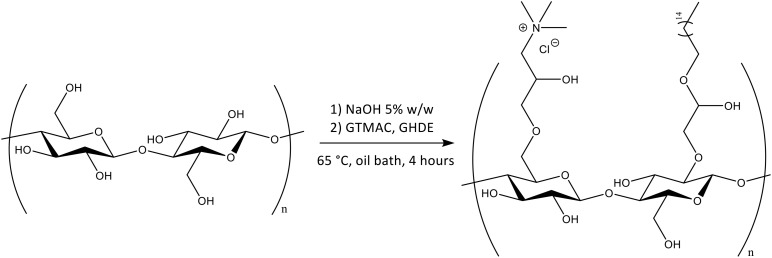
Chemical functionalization of the surface hydroxyl groups of BC by reaction with GTMAC and GHDE.

A ring-opening reaction was carried out under heterogeneous conditions by basic pre-treatment to achieve the deprotonation of the surface hydroxyl groups of cellulose followed by simultaneous addition of the epoxides. First, the hydroxyl groups were deprotonated through basic pre-treatment using 5% w/w NaOH. The active reagents were then added to the reaction mix simultaneously (Figure 3). No macroscopic modifications were observed after the reaction, with the hydrogel retaining the same color and appearance.

### Chemical Characterization

**FIGURE 4:**
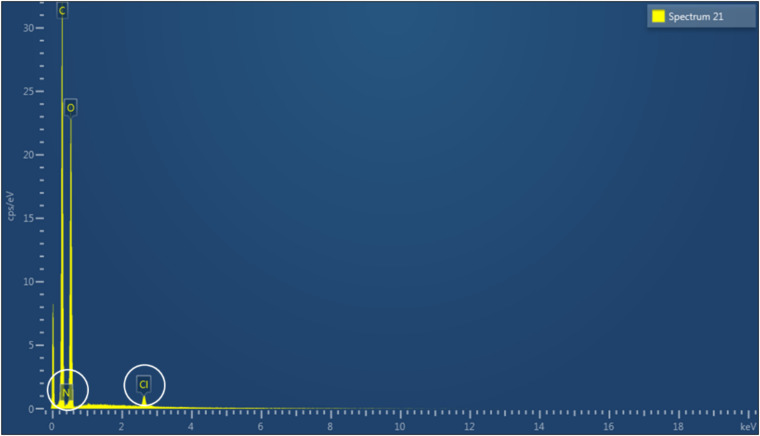
EDX spectrum of modified cellulose.

As cellulose does not present inherent antibacterial properties, the membranes produced were chemically modified in order to achieve this functionality through reaction with two epoxides, namely GTMAC and GHDE. To investigate the degree of substitution, the material was characterized using solid-state techniques due to the insolubility of cellulose in common solvents. First, EDX spectroscopy was performed to confirm the presence of the elements belonging to the functional groups introduced (Figure 4). As carbon and oxygen were already present in the structure of unmodified cellulose, the analysis did not allow to confirm the incorporation of GHDE. However, it was observed that nitrogen and chlorine were successfully introduced as a result of the reaction with GTMAC.

A quantitative indication of the surface elemental composition of the functionalized material was then obtained through XPS spectroscopy (Table 1). The analysis evidenced a significant increase in the nitrogen content (from 0.29 to 2.63 of atomic percent) as a result of the reaction performed. The presence of nitrogen in plain cellulose could be attributed to residual traces of the culture medium or environmental impurities. As regards the amount of carbon and oxygen, their abundance remained almost constant due to their predominance in the material; however, the relative oxygen content in the modified cellulose showed a decrease (from 38.35 to 35.83%), while the amount of carbon remained constant. This result could indicate the successful incorporation of GHDE. The reagent presents in fact long alkyl chains with a carbon to oxygen ratio of 9:1, whereas the ratio in glucose is 1.2:1, which is closer to the C/O ratio obtained for the unmodified sample as compared to the modified one.

**TABLE 1:** XPS quantitative elemental analysis of unmodified and modified cellulose.

	Element	Area	Atomic %
**Non-functionalized cellulose**	O 1s	2386607.93	38.35
	C 1s	1303162.96	61.36
	N 1s	11008.54	0.29
**Functionalized cellulose**	O 1s	2072893.74	35.83
	C 1s	1215029.43	61.54
	N 1s	93556.40	2.63

### Rheological Measurements

The viscoelastic properties of the material upon chemical modification were investigated through rheological studies. The storage and loss moduli (respectively, *G′* and *G″*) for frequency sweep and temperature ramp assays were determined and compared. Both tests were performed in the linear viscoelastic region (LVR).

**FIGURE 5:**
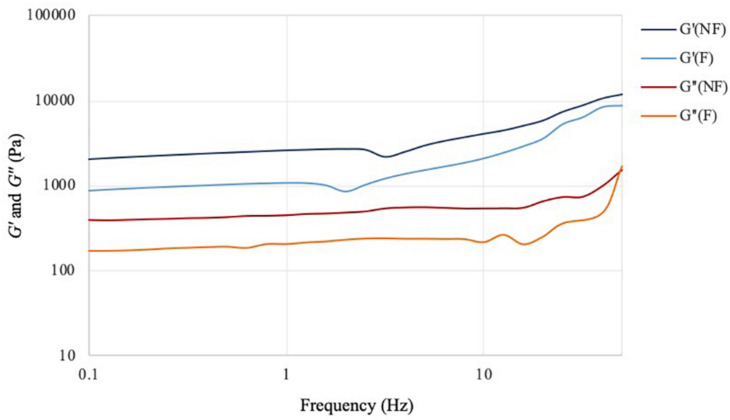
Frequency sweep test of bacterial cellulose before (NF) and after (F) functionalization. *T* = 32°C; preload = 6 Pa.

The frequency sweep analysis (also called small amplitude oscillatory shear, SOAS) showed that both materials behaved as self-standing hydrogels, with the storage modulus (*G′*) higher than the loss modulus (*G″*) at all the frequencies tested (Figure 5). The two materials exhibited similar behavior, with same trend for both dynamic moduli. In particular, *G′* increased proportionally with the frequency, while *G″* presented relatively lower degree of variation throughout the test. This result indicated that the elastic component of the mechanical properties of the material was more affected by the increase in the shear rate, which is typical for hydrogels. Although both samples showed a more elastic-like response to the stimuli, the reaction performed caused a significant decrease of the storage modulus from 2000 Pa for the unmodified sample to almost 900 Pa for the modified one at 0.1 Hz. The same tendency was noted at higher frequencies, where the maximum storage modulus value for the unmodified was about 11,700 Pa vs. ≈9000 Pa for the modified specimen. As regards the viscous component (expressed by the loss modulus), for both hydrogels a linear increase of circa 1000 Pa was observed from 0.1 Hz up to about 35–40 Hz. At higher shear rate, i.e., in the 40–50 Hz region, a higher increase was registered especially in the case of the modified cellulose, with a final *G″* value of about 1570 Pa and 1700 Pa, respectively, for plain cellulose and functionalized cellulose.

**FIGURE 6:**
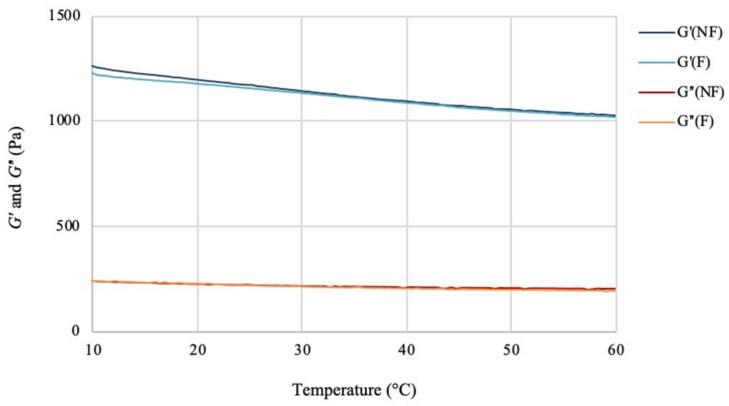
Temperature ramp test of bacterial cellulose before (NF) and after (F) functionalization. *f* = 1 Hz; preload = 6 Pa, rate = 5°C/min.

The effect of the temperature variation on the viscoelastic behavior of the hydrogels was then analyzed by performing a temperature ramp assay in the range between 10 and 60°C (Figure 6). For this test, the two materials showed overlapping values for both storage and loss moduli at all temperatures. As in the case of the frequency sweep assay, the hydrogels presented a solid-like response (*G′* > *G″*) in the range considered. The storage modulus values at 10°C showed minimal decrease for the functionalized sample (1230 Pa) as compared to the non-functionalized one (1260 Pa). The same trend was observed at higher temperatures, with *G′* of about 1030 Pa and 1020 Pa, respectively, for the unmodified and modified pellicles at 60°C. For both samples, a decrease of ≈17–18% in the storage modulus was detected with the increase of temperature, which indicated a more viscous behavior at higher temperatures, as expected in the case of a solid material. However, the presence of a high amount of water (i.e., 97%) did not appear to massively affect the mechanical properties, with the hydrogels retaining a solid-like behavior with good degree of stiffness up to 60°C. The loss modulus values for the two hydrogels showed once again very similar behavior, with only a slight decrease at higher temperatures (about 236–200 Pa for the unmodified and about 237–190 Pa for the modified cellulose), thus indicating that there was no significant effect of the temperature on the viscous component. Overall, the assay confirmed that the functionalization process did not have an adverse impact on the structure and the viscoelastic properties of the material, which maintained a self-standing network in the range of temperatures tested.

### Stability Studies

**FIGURE 7:**
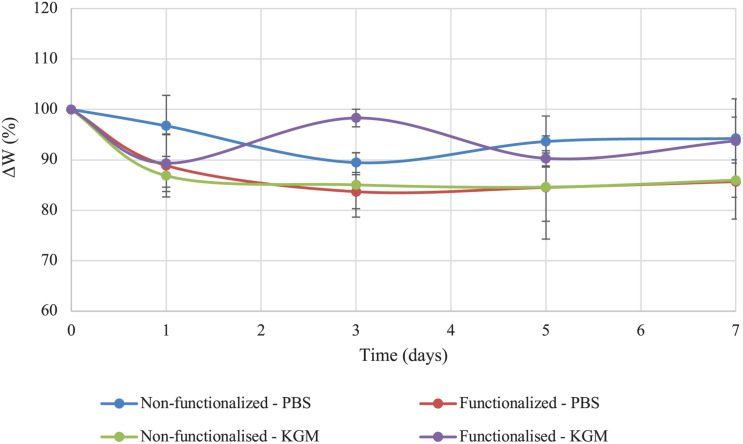
Stability of unmodified and modified bacterial cellulose in PBS solution and keratinocytes growth medium (KGM) at 32°C.

The stability of the pellicles in liquid media before and after the reaction was also investigated. The test was conducted in PBS solution and keratinocytes growth medium (KGM) at 32°C, which has been reported as the average temperature of an acute wound-bed (Fierheller and Sibbald, 2010; Power et al., 2017). The weight variation (ΔW) over 7 days of static incubation in both media was calculated for the two samples and compared (Figure 7).

Both tests indicated that no significant degradation of the material took place in the timeframe considered as a result of the functionalization. As regards the stability in PBS solution, the weight of modified cellulose reached a plateau after about 3 days, with a value of 85% of the initial wet weight that remained constant for up to 7 days upon incubation in the medium. The weight of the unmodified hydrogel, on the other hand, presented a higher degree of variation over time. After 3 days, the sample lost about 10% of the initial weight, and the weight remained almost constant until day 7, with total weight loss of about 5–10%. Overall, the two materials did not show any statistically significant difference at any time point (*p* > 0.05). In the case of incubation in keratinocytes growth medium, the behavior appeared to be reversed, with the non-functionalized sample reaching a plateau in the weight after 3 days (final value: 85% of the initial weight). The weight of the functionalized cellulose decreased by 10% after one day, with a final weight of 90–93% as compared to the initial one. Once again, the two sets of data did not differ significantly for the time points considered, except for day 3 (*p* = 0.01).

### Biological Characterization

#### Antibacterial Assessment

**FIGURE 8:**
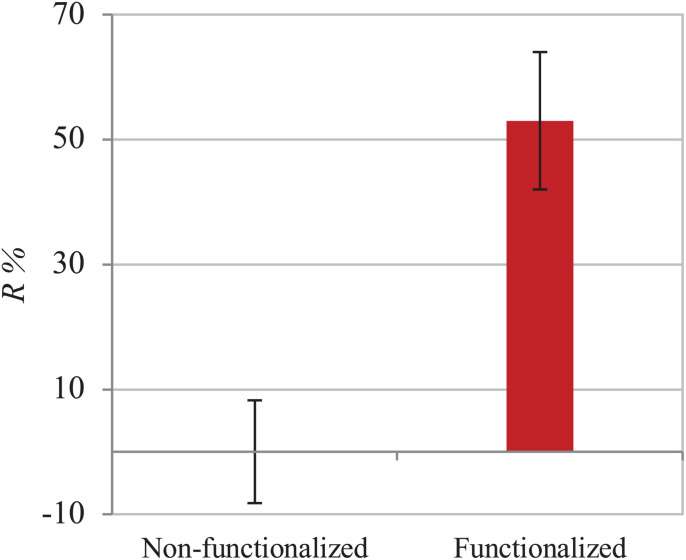
Antibacterial activity of non-functionalized and functionalized BC against *S. aureus.*

The functionalization of the hydroxyl groups of cellulose led to the development of an inherently antibacterial hydrogel with covalently bonded agents, as confirmed by the characterizations carried out after thorough washing of the pellicles to remove any unreacted species. In light of this, the antibacterial properties assessment was conducted through evaluation of the activity upon direct contact with bacteria. The study was carried out following an adapted protocol based on the standard procedure ISO 22196, which involves the quantification of the antibacterial activity with respect to the untreated material (i.e., non-functionalized BC). As indicated in the standard, two strains were used, namely Gram-positive *Staphylococcus aureus* subsp. aureus Rosenbach 6538^TM^ and Gram-negative *Escherichia coli* (Migula) Castellani and Chalmers ATCC^®^ 8739^TM^. The test was first conducted against *S. aureus*. The percent antibacterial activity (*R%*) obtained was calculated as the difference between the number of residual cells on the modified sample and the control divided by the number of residual cells on the control (Figure 8). The value obtained indicated that the bacterial cells growing on the surface of the modified pellicle were reduced by more than half as compared to the unmodified one (i.e., 53%), with a statistically significant reduction of about one order of magnitude in the population in the first 24 h.

**FIGURE 9:**
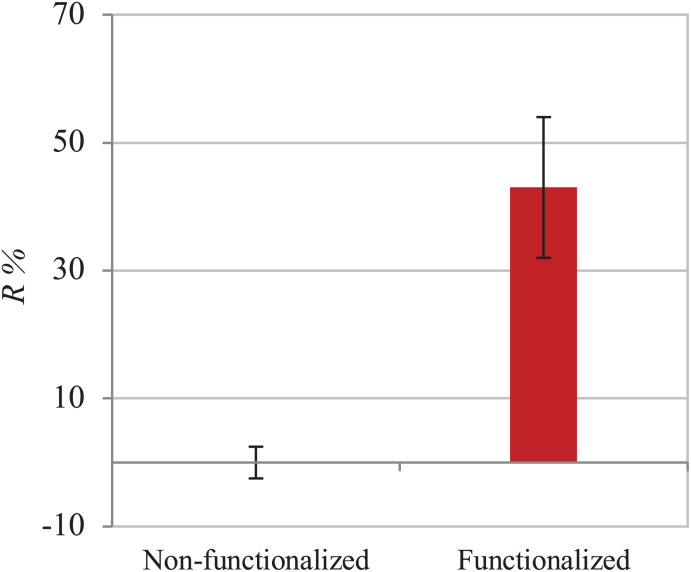
Antibacterial activity of non-functionalized and functionalized BC against *E. coli*.

The assay was then performed using *E. coli* to investigate the effect of the material against Gram-negative strains. The same ISO 22196 procedure was followed to obtain comparable results and the *R%* value was calculated using the same formula (Figure 9). Once again, the number of viable cells decreased by almost half in 24 h as compared to the untreated hydrogel (43%). A statistically significant difference (*p* < 0.05) between the samples before and after functionalization was in fact observed, confirming the effect of the reaction in the successful inhibition of bacterial proliferation.

#### Biocompatibility Studies

##### Cytotoxicity evaluation

**FIGURE 10:**
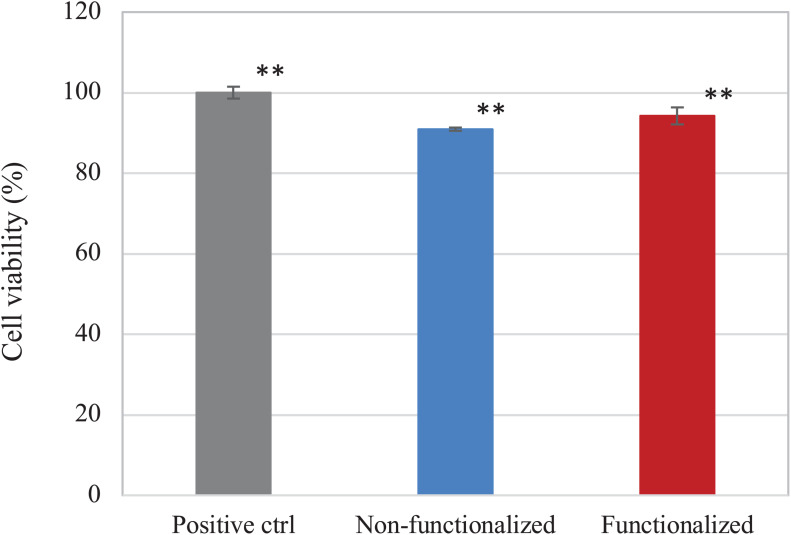
Viability of HaCaT cells upon contact with undiluted eluates from the BC samples. Non-functionalized and functionalized cellulose were found to be significantly different as compared to the positive control, i.e., keratinocytes growth medium (**p < 0.01), whereas no significant difference was observed between the two samples (*p* = 0.06).

The direct and indirect cytotoxicity of the cellulose hydrogels was assessed using keratinocyte human epidermal cells (HaCaT cell line). First, the indirect cytotoxicity was studied to evaluate the presence of leachable products released from the membranes that could cause an adverse effect on the cellular growth (Figure 10). Both samples presented a high degree of biocompatibility, with over 90% cell viability. Furthermore, it was possible to observe that the reaction carried out did not introduce any leachable compound that could have a cytotoxic effect on keratinocytes, as no statistically significant difference was observed between the unmodified and the modified pellicle.

**FIGURE 11:**
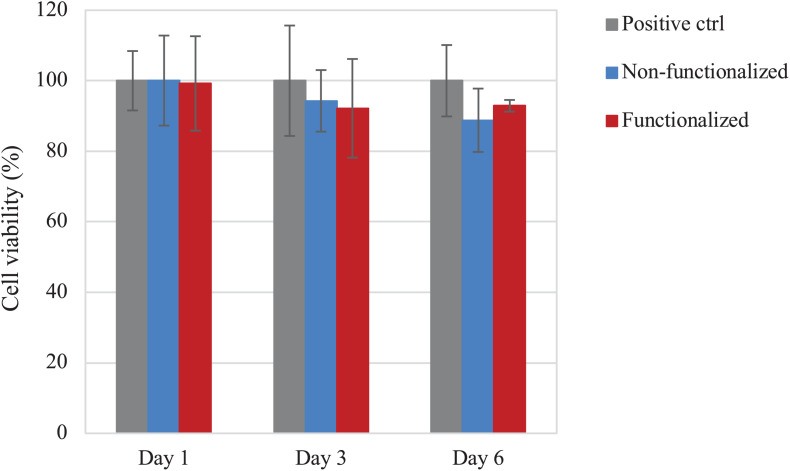
Viability of HaCaT cells upon direct contact with the BC samples. No statistically significant difference was observed between both cellulose samples and the positive control (i.e., tissue culture plastic, TCP) at any time point (*p* > 0.05).

The direct cytotoxicity of the materials was then evaluated following the standard ISO 10993-5 for the biological characterization of medical devices. The cell viability was evaluated after 1, 3, and 6 days of direct contact with the samples (Figure 11). Once again, cell viability values of at least 90% as compared to the positive control were registered for the hydrogels at all timepoints. After 1 day, both samples presented ≈100% cell viability, with no statistically significant difference with the positive control (*p* > 0.05), whereas on day 3 the viability was found to be in the range of 90–95% (94 and 92% for the unmodified and modified pellicles, respectively). After 6 days of incubation with the samples, the cell viability was almost 90% for the neat cellulose and about 93% for the functionalized one. Overall, although the average values of cell viability in the presence of functionalized and, especially, non-functionalized BC seem to decrease over time, no statistically significant difference was observed between the two materials and the positive control for up to 6 days of direct contact with the cells (p >> 0.05 for non-functionalized and functionalized BC as compared to the positive control at all time points).

**FIGURE 12:**
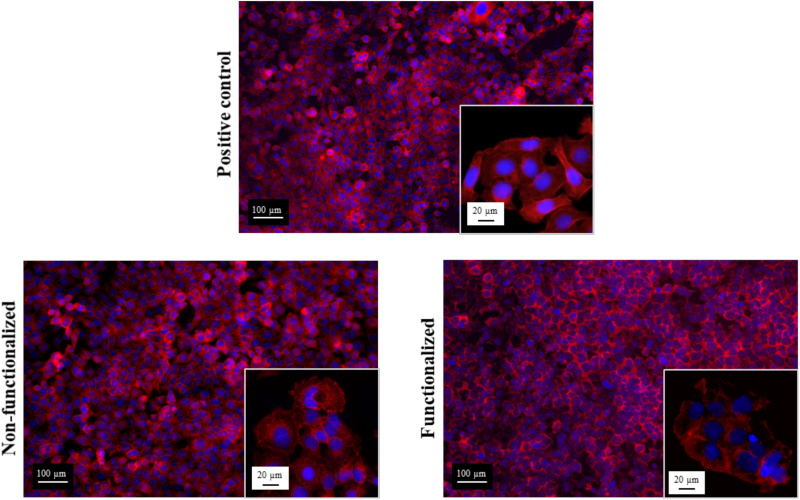
Confocal microscopies of keratinocytes after 6 days of incubation on the bacterial cellulose stained using phalloidin (red) and DAPI (blue).

In addition to this, the morphology of the cells after 6 days of contact with the hydrogels was evaluated through fluorescent staining of the nuclei and the actin filaments using, respectively, DAPI and phalloidin, and was compared to the control group (Figure 12).

All the images confirmed the formation of a homogeneous monolayer of adherent keratinocytes. No qualitative difference in the morphology was observed between the three groups, confirming that the cellulose hydrogels supported the growth and proliferation of the cells. In particular, a polygonal morphology typical of keratinocytes was observed, with the cells arranged in a “cobblestone” pattern. The nuclei appeared to be spherical, surrounded by a homogeneous cytoplasmic region. The size and structure of the cells was found to be the same for the ones in contact with the samples and the positive control, with an average diameter of about 15–20 μm.

##### In vitro scratch assay

**FIGURE 13:**
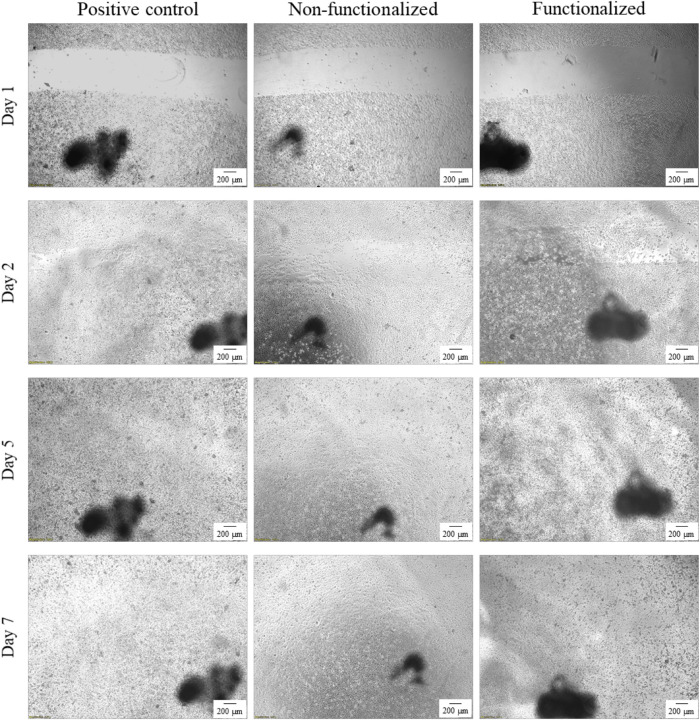
Visual progression of the coverage of the scratched area by cells over time, until wound closure (40×). The black mark in the images is the reference for the scratch.

**FIGURE 14:**
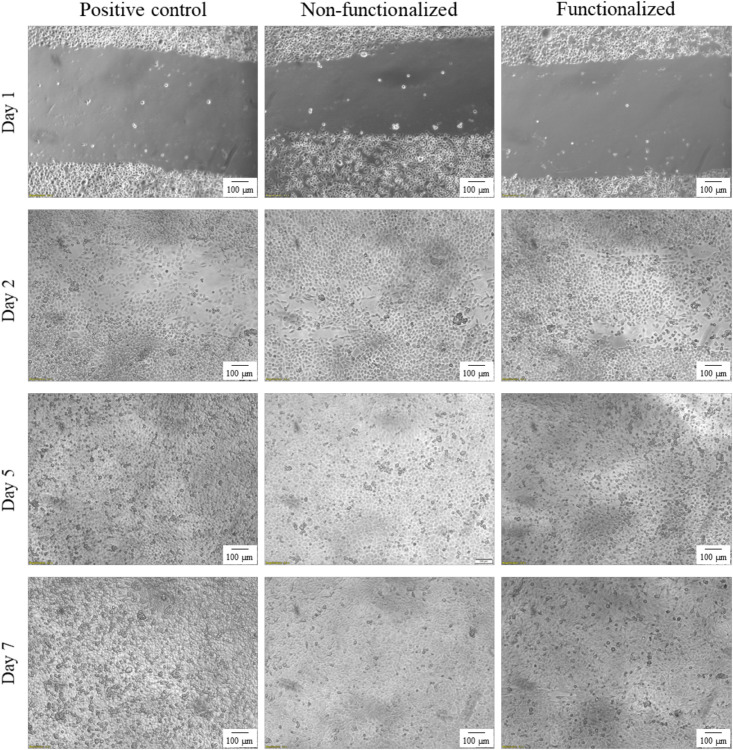
Visual progression of the coverage of the scratched area by cells over time, until wound closure (100×).

An *in vitro* scratch assay was carried out using immortalized keratinocyte cells obtained from histologically normal skin in order to investigate the wound healing process in the presence of the hydrogels. This test allows in fact to assess the cell mobility without and in the presence of cellulose samples through creation of a scratch in the cell monolayer. The migration and proliferation of the cells over time was then evaluated until wound closure. In this study, the wound margins were observed over 7 days until complete coverage of the scratch (Figures 13, 14). The images showed that for all the groups almost complete closure was reached within 5 days. The presence of the two hydrogels did not inhibit cell migration to the wound area, and the recovery rate was found to be very similar for the two hydrogels and the control, i.e., keratinocytes growth medium only. After 2 days, the scratched area started to be covered with keratinocytes, proving that the cells were alive and active from both migratory and proliferative point of view.

**FIGURE 15:**
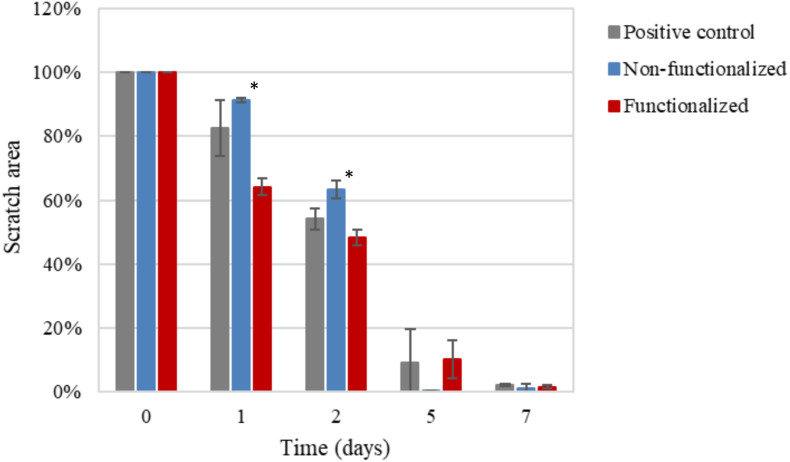
Progression of the closure of the scratch area over time as the ratio between black pixels at time × and black pixels at time 0 (set as 100%, i.e., no coverage by cells). The non-functionalized cellulose group was found to be significantly different as compared to the positive control, i.e., keratinocytes growth medium, at day 1 and 2 (*p < 0.05), whereas no significant difference was observed between the functionalized cellulose and the control at any time point.

After a first qualitative assessment, the images were analyzed in order to quantitatively evaluate the rate of wound closure in the presence of unmodified and modified cellulose and in comparison with the control. The percent of the scratched area covered by cells over time was quantified using an image processing software by considering the extension of the area covered by cells (white pixels) and the area not covered by cells (black pixels) (Figure 15). As expected, the calculations confirmed that the three groups showed similar behavior and wound progression trend, with a linear decrease of the scratch and almost complete coverage by the cells within 5 days. In particular, for the unmodified cellulose wound closure of 100% was reached, whereas both the modified and the positive control presented a value of ≈90%. The statistical analysis carried out for the values obtained at each time point showed no significant difference between the functionalized hydrogel and the control at any time point. A similar trend was observed for the non-functionalized sample, although this was found to significantly differ from the control on day 1 and day 2 (*p* = 0.03 and *p* = 0.04, respectively).

## Discussion

In this study, a water-based green method for the surface modification of bacterial cellulose was proposed to impart antibacterial activity. To achieve so, BC pellicles were produced through static fermentation of *Gluconacetobacter xylinus* in the presence of glucose as the main carbon source. More specifically, a modified HS medium was used, which consisted of glucose, yeast extract and CaCO_3_. Although several sugars such as mannitol, sucrose and fructose have been successfully used as carbon sources, it was observed that glucose resulted in higher yield under the conditions applied in this study. Similarly, yeast extract was found to give higher yield as compared to other nitrogen sources, including corn steep liquor and beef extract (Son et al., 2001; Jia et al., 2004; Castro et al., 2012). Finally, calcium carbonate was chosen as the metal ions source, as recent studies evidenced that the introduction of ions such as calcium and magnesium results in an increase of the polymer production yield (Son et al., 2003; Hungund and Gupta, 2010; Hong et al., 2011).

The heterogeneous reaction was then conducted in one step upon deprotonation of the hydroxyl groups of the glucose units in the presence of sodium hydroxide. The derivatization of the hydroxyl groups was carried out through a base-catalyzed ring-opening reaction of two epoxides involving the alkylation of the oxygen, thus resulting in the etherification of the hydroxyl groups. The reaction is believed to proceed as a regioselective nucleophilic attack (probably via a S_N_2 mechanism) of the oxygen to the less hindered electrophilic carbon of the epoxide ring (Padwa and Murphree, 2000; Smith, 2010). The functionalization was performed using water as the solvent, both because of the high original water content of the substrate, as this polysaccharide is naturally biosynthesized as a hydrogel with a minimum water content of 97% (even higher values might be obtained through harsher drying techniques such as freeze-drying or oven-drying), as well as to ensure that no traces of toxic volatile solvents that could alter the crystalline organization of the fibrils would remain in the network. Due to the intrinsic insolubility of cellulose in most solvents, solid-state techniques were used to characterize the modified material. In particular, EDX and XPS were carried out on the hydrogel before and after functionalization to evaluate the degree of substitution. The presence of the nitrogen and chlorine peaks in the EDX spectrum indicated successful incorporation of GTMAC. In addition to this, the increase in the nitrogen content as well as the decrease in the superficial carbon to oxygen ratio detected by the XPS further confirmed the attachment of both GTMAC and GHDE. As compared to the EDX analysis, however, XPS did not detect chlorine in the area considered. This result might be related to the ability of XPS to give information only for the most external layer of the sample, i.e., 0.8–5 nm, whereas in the case of EDX the elemental composition of the outer 0.2–8 μm region can be observed (Walzak et al., 1998). It is possible, in fact, that chlorine (a counterion) was not visible as it had been displaced before/during the analysis due to the non-covalent nature of the bond with the quaternary ammonium. In addition to this, it is also possible that the XPS analysis did not detect chlorine in the area of analysis due to the inhomogeneity of the reaction, which was conducted under heterogeneous conditions. Nevertheless, the analyses showed that the epoxides were permanently bonded to the polymer backbone, as the washing procedure to which the pellicles were subjected prior to the characterization did not affect the result. This finding is particularly important with respect to the antibacterial properties of the dressing. A permanent modification of the structure can in fact ensure long-term activity against bacteria, as opposed to the physical absorption of leachable agents, which is limited by the maximum loading amount that can be incorporated into the matrix and released over time (Poverenov et al., 2013; Giano et al., 2014).

The effect of the reaction on the mechanical properties of BC was investigated through rheological studies. The storage and loss moduli (respectively, *G′* and *G″*) of the hydrogels were measured through frequency sweep and temperature ramp assays. As regards the frequency sweep assay, similar trends were observed for the two samples, with increasing storage modulus as a response to increased oscillation rate and lower degree of variation for the loss modulus in the interval of frequencies applied. This is a common feature for hydrogels, as they typically present a more solid-like response at increased frequencies, i.e., higher stiffness upon fast movement in a short time-scale (Sanabria-DeLong et al., 2007; Yan and Pochan, 2010). The results obtained for the functionalized sample evidenced a slight decrease in the storage and loss moduli. The effect could probably be ascribed to the high temperature and basic treatment required for the reaction, which might have caused a certain degree of degradation due to alkaline hydrolysis of the polymer (Shibazaki et al., 1997; Reiniati et al., 2016). Nevertheless, the hydrogel presented a predominantly elastic response with *G′* > *G″* at all the conditions tested. Similar results were observed for the temperature ramp test, with only 1–2% variation for both dynamic moduli even at higher temperatures. Overall, the membranes presented good mechanical properties as compared to previously published results based on cellulose hydrogels as well as synthetic polymers-based substrates. It was found, in fact, that most hydrogels exhibit a more viscous behavior at temperatures over 35–40°C, probably due to the degradation of the crystalline regions that results in the disruption of the 3D-network (Van Den Bulcke et al., 2000; Huang et al., 2010; Roy et al., 2010; Smith et al., 2010; Zuidema et al., 2014). This effect was not observed in this study, as the membranes retained a solid structure and good stiffness even at 60°C. This is a crucial feature for wound healing applications, as a dressing needs to be easy to handle and to remove (for dressing change), stiff enough to sustain the cell growth and the tissue surrounding the wound while able to adapt to its shape (Boateng et al., 2008; Kocen et al., 2017). Furthermore, a stability study was conducted on BC in order to assess the degradation of the pellicles in liquid media in terms of weight loss. The test did not evidence major degradation over 7 days, as both samples retained circa 85–90% of their initial weight in PBS solution and keratinocytes growth medium with no statistically significant difference between them except for one time point.

Finally, the hydrogels were characterized from the biological point of view to assess both their antibacterial properties and their biocompatibility. In particular, as the agents were covalently bonded to the backbone of the polymer, the antibacterial activity was studied following a direct contact protocol. For both *S. aureus* and *E. coli*, the functionalized material caused a decrease by circa half in the cell count as compared to plain cellulose. More specifically, a 53% reduction was observed for *S. aureus* vs. 43% in the case of *E. coli*. This difference in the activity can be ascribed to the structure of the cell membrane of Gram-negative bacteria as well as the mechanism of action of the groups involved. Gram-negative strains possess an outer membrane in addition to the peptidoglycan layer. This membrane consists of a lipid bilayer containing phospholipids and glycolipids and acts as a permeability barrier, making the cell less susceptible to the penetration of biocidal agents (Silhavy et al., 2010; Malanovic and Lohner, 2016; Miller, 2016; Exner et al., 2017). Due to this additional layer, Gram-negative bacteria are considered a greater threat in the medical field since they show higher degree of resistance toward traditional antibiotics. In this context, few substances have been found to be active against Gram-negative bacteria. Among these, quaternary ammonium groups have demonstrated to act against a broad spectrum of microorganisms thanks to their non-selective mechanism of action. As mentioned in the introduction, it is commonly acknowledged that the biocidal activity of these compounds is related to the cationic nature of the nitrogen, which is able to electrostatically interact with the negative charges of the bacterial cell membrane. Zeta potential and electrophoretic mobility studies on *E. coli* and *S. aureus* showed in fact a net negative charge on the bacterial cell envelope, although higher values were registered for *E. coli* (Sonohara et al., 1995; Halder et al., 2015). These results indicated that the presence of the additional negatively charged layer in Gram-negative strains caused an increase in the anionic potential of the membrane, suggesting that achieving a higher cationic charge density on the polymer might have a stronger impact against such bacteria (Silhavy et al., 2010). In addition to this, the alkyl chains attached to the backbone probably contributed to the overall antibacterial activity thanks to their lipophilic nature. It has been proven that such groups can penetrate through the lipid regions of the membrane through hydrophobic interactions, thus causing its disruption with consequent leakage of intracellular components. In particular, the activity was found to be dependent on the chain length, with the optimal carbon chain in the range between C_12_ and C_14_ (Kubo et al., 1993; Wiener and Horanyi, 2011; Zheng et al., 2016). In light of the results obtained, it is also worth mentioning that an increase in the effectiveness of the reaction would probably lead to higher antibacterial action. It has been reported that the presence of water can decrease the degree of substitution in ring-opening reactions due to the hydrolysis of the alkylating agent under basic conditions (Zaman et al., 2012). This effect, together with the solid-liquid reaction kinetics, could have caused an anisotropy in the bactericidal activity. It is in fact possible that the heterogeneous conditions applied led to the non-homogeneous distribution of the functional groups, causing some differences in the surface structure and, therefore, the antibacterial effect (Ramsden, 2011; Salmi et al., 2013).

The biocompatibility of the modified hydrogel was studied using the human epidermal keratinocyte cell line (HaCaT) and compared to pristine BC. First, the cytotoxicity upon indirect contact was evaluated to investigate the presence of residual leachable compounds that could cause an adverse reaction toward cells. A direct cytotoxicity assay was then carried out over a period of 6 days, as the average time between two consecutive dressing changes has been reported as 1–3 days (Purser, 2010; Sood et al., 2014). In all cases, cell viability of about 90% was obtained for both materials, with no statistically significant difference between BC and the positive control at any timepoint. The results obtained were in line with previously published literature on the cytotoxicity of various polymers derivatized with quaternary ammonium compounds. No inhibitory effect was in fact observed toward various types of cells including fibroblasts and human dental pulp cells, confirming the biocompatibility of such groups toward eukaryotic cells even when compared with well-established antibacterial agents such as silver (Ding et al., 2009; Li et al., 2013; Huang et al., 2018). After 6 days, the morphology of the cells was visually assessed through staining of the nuclei and the cytoplasm using DAPI and phalloidin. In all cases, the presence of an adherent monolayer of cells was observed, with the typical polygonal morphology of keratinocytes and a “cobblestone” growth pattern (Koehler et al., 2011). The size of the cells was also in accordance with previous studies. The diameter was in fact in the range of 15–20 μm, which has been indicated as the average size for young keratinocyte cells (Sun et al., 2007; Soroka et al., 2008). In addition to this, it is possible that a certain degree of penetration of the cells into the polymer matrix occurred. Previous works evidenced in fact the ability of BC to support the infiltration of cells throughout the third dimension of the scaffold, in a proportional manner with respect to the cells size and the pore size of the nanofibrillar structure (Luo et al., 2019; Ul-Islam et al., 2019b).

An *in vitro* scratch assay was also carried out in the presence of the cellulose-based hydrogels using keratinocyte cells. Despite the functionalization performed, the quantitative assessment of the scratch coverage by the cells evidenced no statistically significant difference between the modified cellulose and the control at any of the timepoints considered. In addition to this, the wound closure rate was found to be in agreement with the literature: after only 12 h, the wound started to be re-populated, showing a pattern of growth comparable with previously published research. Studies on the mechanism of wound healing indicated in fact that keratinocytes start migrating after 6–24 h upon wounding and express keratin after 8–24 h. This is a crucial step in the healing process, as cell migration is the rate-limiting event, with keratinocytes presenting lower migration rates as compared to dermal fibroblasts. In particular, fibroblasts are believed to be the first type of cells that migrate to the wound site together with endothelial cells, followed by keratinocytes that induce re-epithelialization. Finally, a remodeling stage takes place involving a second migration of fibroblasts (Usui et al., 2008; Walter et al., 2010). These encouraging findings prove that the reaction carried out induced an adverse effect against bacteria without affecting the wound healing process, as the cells presented good migratory ability in the short term as well as active proliferative behavior after various days of contact with the samples.

## Conclusion

In this study, for the first time, a one-step method for the modification of never-dried bacterial cellulose was developed in order to introduce antibacterial features. The procedure proposed included a green-chemistry approach for the derivatization of the hydroxyl groups of glucose without using any toxic organic solvent. The functionalization was in fact carried out in water through base-catalyzed heterogeneous reaction with two different epoxides, without affecting the hydrogel structure of the biopolymer. The material was then chemically characterized to investigate the incorporation of the active groups. The viscoelastic behavior before and after the modification was also studied to assess the mechanical properties of the hydrogels. The tests highlighted that no major degradation occurred as a result of the chemical treatment, with very similar storage and loss moduli for both the frequency sweep and the temperature ramp assays. The stability studies confirmed these findings, as the samples retained good structural features for up to 7 days of incubation in liquid media. A biological evaluation was conducted to investigate the antiproliferative ability and cytotoxicity of the samples. First, the activity upon direct contact with *S. aureus* and *E. coli* was determined, and for both strains a reduction by circa half in the bacterial cell population was observed within 24 h in the case of the functionalized material as compared to the unmodified one. In addition to this, cytotoxicity studies evidenced that no adverse effect was caused by the cellulose hydrogels upon indirect or direct contact with keratinocytes even after 6 days of contact. The staining of the cells, in fact, did not reveal any difference in the morphology, growth pattern and size of the cells in the three groups. Finally, the *in vitro* scratch assay highlighted that the presence of the samples did not interfere with the healing process, with wound closure rates comparable to the control. Future works can focus on better characterizing the material developed from the chemical point of view, for instance by performing a solid-state NMR for the quantitative evaluation of the reaction yield as well as by assessing the elemental distribution related to the functional groups introduced in the structure. In addition to this, the mechanical properties of the modified pellicles can be further investigated with respect to their application as wound dressings, for instance through compressive dynamic mechanical analysis (DMA) and nanoindentation.

Overall, this work represents a promising strategy for the use of bacterial cellulose as a wound dressing platform with tailorable properties. Its innate hydrogel-like nature can in fact provide a high level of moisture to dry wounds and great biocompatibility, while the functionalization carried out can ensure an inhibitory effect against both Gram-positive and Gram-negative bacteria through an environmentally friendly procedure. Furthermore, this versatile methodology can also be applied to other polysaccharides such as hyaluronic acid and chitosan to introduce inherent antibacterial features or improve their efficiency.

## Data Availability Statement

All datasets generated for this study are included in the article/supplementary material.

## Author Contributions

IO performed the experiments, analyzed the data, and wrote the manuscript. PB developed and optimized the BC production method. RN contributed to the reaction design. WW supervised the scratch assay performed in this work. JCK was involved in the SEM/EDX and confocal microscopy. IR supervised the experimental work and corrected the manuscript. All authors contributed to the article and approved the submitted version.

## Conflict of Interest

The authors declare that the research was conducted in the absence of any commercial or financial relationships that could be construed as a potential conflict of interest.
